# Generalized Gaussian Distribution Improved Permutation Entropy: A New Measure for Complex Time Series Analysis

**DOI:** 10.3390/e26110960

**Published:** 2024-11-07

**Authors:** Kun Zheng, Hong-Seng Gan, Jun Kit Chaw, Sze-Hong Teh, Zhe Chen

**Affiliations:** 1 Institute of Visual Informatics, National University of Malaysia (UKM), Bangi 43600, Selangor, Malaysia; raineejinn@gmail.com; 2 School of Information and Communication, Guilin University of Electronic Technology, Guilin 541004, China; chenzhe@mail.nwpu.edu.cn; 3 School of AI and Advanced Computing, XJTLU Entrepreneur College (Taicang), Xi’an Jiaotong—Liverpool University, Suzhou 215400, China; hongseng.gan@xjtlu.edu.cn; 4 School of Intelligent Manufacturing Ecosystem, XJTLU Entrepreneur College (Taicang), Xi’an Jiaotong—Liverpool University, Suzhou 215400, China; 5 Cognitive Radio and Information Processing Key Laboratory Authorized by China’s Ministry of Education Foundation, Guilin University of Electronic Technology, Guilin 541004, China

**Keywords:** improved permutation entropy, feature extraction, data analysis

## Abstract

To enhance the performance of entropy algorithms in analyzing complex time series, generalized Gaussian distribution improved permutation entropy (GGDIPE) and its multiscale variant (MGGDIPE) are proposed in this paper. First, the generalized Gaussian distribution cumulative distribution function is employed for data normalization to enhance the algorithm’s applicability across time series with diverse distributions. The algorithm further processes the normalized data using improved permutation entropy, which maintains both the absolute magnitude and temporal correlations of the signals, overcoming the equal value issue found in traditional permutation entropy (PE). Simulation results indicate that GGDIPE is less sensitive to parameter variations, exhibits strong noise resistance, accurately reveals the dynamic behavior of chaotic systems, and operates significantly faster than PE. Real-world data analysis shows that MGGDIPE provides markedly better separability for RR interval signals, EEG signals, bearing fault signals, and underwater acoustic signals compared to multiscale PE (MPE) and multiscale dispersion entropy (MDE). Notably, in underwater target recognition tasks, MGGDIPE achieves a classification accuracy of 97.5% across four types of acoustic signals, substantially surpassing the performance of MDE (70.5%) and MPE (62.5%). Thus, the proposed method demonstrates exceptional capability in processing complex time series.

## 1. Introduction

The primary objective of complex time series analysis is to classify and identify signals by extracting their distinctive features or patterns. To perform this task, entropy-based methods, which enhance the understanding of underlying dynamics, have garnered more and more attention in recent years [1,2]. These methods have been successfully applied across various complex engineering domains, including electrocardiogram (ECG) and electroencephalographic (EEG) records analysis [3,4,5], bearing fault diagnosis [6], and underwater target recognition [7,8].

The prevalent entropy metrics can be broadly classified into two main categories: those that are conditional entropy-based and those that are Shannon entropy-based. The former includes widely used methods such as Approximate Entropy (ApEn) [9], Sample Entropy (SampEn) [10], and Fuzzy Entropy (FuzzyEn) [11]. The core of these measures is to compare the probabilities of similar patterns occurring in m dimensional and m+1 dimensional phase spaces to infer the emergence of new patterns. Pattern similarity is determined by whether the distance between vectors falls below a predefined threshold. For example, if $D\left(X_{i}^{m},X_{j}^{m}\right)\leq r$ and $D\left(X_{i}^{m+1},X_{j}^{m+1}\right)>r$, where $D\left(x,y\right)$ represents the distance between x and y, and r is a constant, it suggests the emergence of a new pattern when the embedding dimension is enlarged from m to m+1. Generally, the more components and complexity within a time series, the higher the probability of new patterns emerging, resulting in a higher entropy value. The main drawback of the aforementioned methods lies in their requirement for extensive distance calculation within the phase space, which results in high computational complexity. Furthermore, when dealing with short data sequences, these methods are prone to generate undefined entropy values.

Without being exhaustive, Shannon entropy-based approaches include Permutation Entropy (PE) [12], Distribution Entropy (DistEn) [13], Weighted Permutation Entropy (WPE) [14], Modified Permutation Entropy (mPE) [15], Dispersion Entropy (DispEn) [16], Slope entropy (SlopEn) [17], and Attention Entropy (AE) [18], among others. Permutation entropy, introduced by Bandt et al. [12] estimates probability density based on the frequency of ordinal patterns (OP). Its conceptual simplicity and computational efficiency have quickly made it one of the most prominent entropy algorithms [2,17,19,20]. Subsequently, WPE was proposed to address the limitation of PE in neglecting absolute amplitude information. In WPE, weights are assigned to each OP to calculate the weighted probability density [14]. Bian and the coauthors addressed the distortion in PE estimation that can occur under low-resolution conditions. They accomplished this by mapping equal values within sub-sequences to the same symbol and then calculating the probability distribution of the resulting symbolic patterns [15]. In 2016, Rostaghi et al. [16] developed DispEn, which estimates entropy by calculating the probability density of newly designed symbolic patterns known as dispersion patterns. Compared to PE and its variants, DispEn demonstrates a superior ability to distinguish complex time series [16,19,20]. In 2019, Cuesta-Frau invented SlopEn, which utilizes a new encoding technique based on the gradient between two consecutive sample points [17]. However, a key challenge with SlopEn is the lack of theoretical guidance for determining certain parameters, which may affect the algorithm’s effectiveness. Unlike PE, DispEn, and SlopEn, AE exclusively focuses on the frequency distribution of intervals between key observations in a time series, such as the intervals between local maxima [18]. This parameter-free approach demonstrates promising potential in the analysis of heart rate signals. In summary, the primary differences among these Shannon entropy-based algorithms are rooted in their approaches to estimating the probability density distribution of time series.

Entropy metrics are still continually evolving, with researchers exploring various strategies to enhance their capacity to analyze complex time series. Recognizing that real-world data often exhibit intricate structures across multiple temporal scales, Costa et al. proposed integrating entropy quantification methods with coarse-graining techniques, leading to the development of Multiscale Entropy (MSE) [21]. This innovative approach has been widely adopted by scholars and has become a vital tool for analyzing practical engineering data [22,23]. Additionally, some researchers have developed complexity-entropy plane algorithms [24,25,26,27,28,29,30,31,32,33], which map time series onto a two-dimensional plane as points or curves, thereby improving the discriminability of entropy features. However, it is important to note that the effectiveness of both Multiscale Entropy and complexity-entropy plane approaches is still influenced by the choice of entropy measure employed.

This highlights that traditional entropy algorithms possess unique advantages and limitations. The effectiveness of newer methods, such as DispEn, SlopEn, and AE, in various complex engineering applications has not yet been fully established. To address this gap, this paper presents a new entropy measure known as Generalized Gaussian Distribution Improved Permutation Entropy (GGDIPE). Firstly, the generalized Gaussian distribution cumulative distribution function (GGDCDF) with a specific shape parameter β is employed for data normalization. This technique ensures that our method is adaptable to a range of time series with different underlying distributions. Secondly, a new encoding approach for generating symbolic patterns is presented. This strategy combines the strengths of SampEn, PE, and its modifications, effectively preserving both temporal correlation and absolute amplitude information. Moreover, it resolves the issue of equal values that is encountered in PE. Finally, the probability density of our newly designed symbolic patterns is calculated, and the GGDIPE is derived based on Shannon entropy. To enhance the proposed algorithm’s capability in handling complex time series, this paper also introduces a multiscale version of GGDIPE that utilizes the coarse-graining technique.

The remainder of this paper is organized as follows: a detailed description of GGDIPE is provided in Section 2; synthetic and experimental data processing results are demonstrated and discussed in Section 3 and Section 4, respectively; the paper is concluded in Section 5.

## 2. GGDIPE

**Figure 1:**
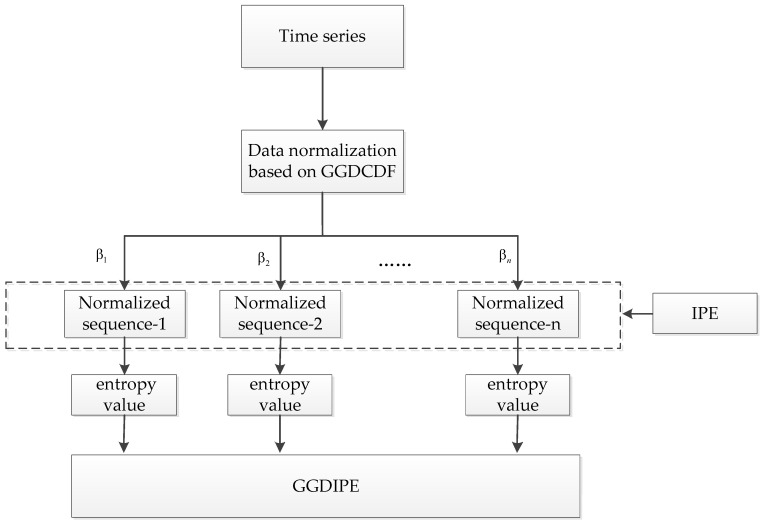
Calculation flow chart of generalized Gaussian distribution improved permutation entropy (GGDIPE).

As illustrated in Figure 1, the proposed GGDIPE is developed through a series of steps: data normalization, improved permutation entropy (IPE) calculation, and the integration of entropy feature vectors. Each of these processes is described in detail in the following subsections. Additionally, a numerical example is provided in Appendix B to enhance clarity.

### 2.1. Data Normalization

The benefits of data normalization are well established in numerous engineering applications. DispEn’s strong data processing capabilities can be partially attributed to its use of the Gaussian cumulative distribution function for normalization. However, real-world data often deviates from Gaussian distributions. To address this issue, the GGDCDF with various shape parameters are employed for data normalization in this paper. This approach ensures that our method is adaptable to a wide range of time series with different underlying distributions.

As shown in Equation (1), given a shape parameter β and a time series $x=x_{1},x_{2},x_{3}\cdots ,x_{N}$, the GGDCDF will transform x into a new sequence y, where the elements of y range from 0 to 1.

(1) $$y=\frac{1}{2}+\mathrm{sgn}(x-\mu )\frac{\overset{\wedge }{\gamma }\left[1/\beta ,{\left(\frac{\left|x-\mu \right|}{\rho }\right)}^{\beta }\right]}{2\Gamma (1/\beta )}$$

In Equation (1), sgn represents the Sign Function, as shown in Equation (2).

(2) $$\mathrm{sgn}(x)=\left\{\begin{array}{c} -1\;\mathrm{if}\;x<0\\ 0\;\mathrm{if}\;x=0\\ 1\;\mathrm{if}\;x>0 \end{array}\right.$$

μ denotes the average of x; Γ stands for the famous gamma function; $\rho =\sigma \sqrt{\left(\Gamma (1/\beta )/\Gamma (3/\beta )\right)}$, with σ the standard deviation of x; and $\overset{\wedge }{\gamma }$ denotes the lower incomplete gamma function. Obviously, an element $y_{i}$ represents the probability that a random variable from the Generalized Gaussian Distribution (GGD) is less than $x_{i}$. The GGD can be tailored to different forms by varying the shape parameter β. Specifically, when β=2, the GGD approximates a normal distribution, whereas when β=∞, it transforms into a uniform distribution [34]. A brief introduction to GGD can be found in Appendix B.

### 2.2. IPE

The IPE employs a new encoding strategy for generating symbolic patterns. Let $y=y_{1},y_{2},y_{3}\cdots ,y_{N}$ be the normalized time series under $\beta_{i}$, the data is first segmented into several m dimensional vectors, with the *j*-th vector denoted as $Y(j,:)=[y_{j},y_{j+1},\cdots y_{j+(m-1)}]$, j=1,2,⋯N−m+1. This process is also known as phase space reconstruction, and m is called the embedding dimension.

To incorporate the absolute amplitude information of y, the first element of the *j*-th m dimensional vector Y(j,1) is symbolized using a Linear partition operator (LPO), as shown in Equation (3), yielding S(j,1). Herein, $y_{\min}$ and $y_{\max}$ represent the minimum and maximum values of y, respectively. L corresponds to the number of symbols used to characterize the time series, and $\Delta =(y_{\max}-y_{\min})/L$. In fact, the role of L in our algorithm is analogous to the tolerance r in SampEn. In SampEn, two vectors are considered similar if $D\left(X_{i}^{m},X_{j}^{m}\right)\leq r$. Similarly, in our algorithm, Y(i,1) and Y(j,1) are assigned the same symbol if they fall within the same region. For example, if $y_{\min}\leq Y(i,1)<\Delta +y_{\min}$ and $y_{\min}\leq Y(j,1)<\Delta +y_{\min}$, the symbol 0 will be assigned to both Y(i,1) and Y(j,1). As will be shown in Section 3, a smaller L increases information loss during symbolization, reducing the precision of entropy estimation but enhancing noise resistance. Conversely, a larger L minimizes information loss and leads to more accurate entropy estimation. In summary, selecting L requires a balance between the accuracy of entropy estimation and the robustness to noise.

(3) $$\mathrm{LPO}(y)=\left\{\begin{array}{cc} 0 & y_{\min}\leq y<\Delta +y_{\min}\\ 1 & y_{\min}+\Delta \leq y<2\Delta +y_{\min}\\ \vdots & \vdots \\ L-1 & y_{\max}-\Delta <y\leq y_{\max} \end{array}\right.$$

To take into account the temporal correlation information, the *k*-th (2≤k≤m) element of the *j*-th vector, represented by Y(j,k), is symbolized by Equation (4), yielding S(j,k). The function ⌊ ⌋ returns the largest integer less than or equal to the input. Evidently, S(j,k) is derived from S(j,1) and the difference between Y(j,k) and Y(j,1). Similar to OP, temporal correlation information is encoded into S(j,k) through comparing the two points.

(4) $$S(j,k)=S(j,1)+\left\lfloor (Y(j,k)-Y(j,1))/\Delta \right\rfloor$$

After symbolization, each m dimensional vector is mapped to a symbol pattern, denoted as S(j,k), 1≤k≤m, j=1,2,⋯N−m+1. There are $L^{m}$ underlying symbol patterns in the IPE algorithm. The probability of each symbol pattern $p_{i}$($1\leq i\leq L^{m}$) is then counted, and the IPE is finally calculated using Equation (5), where $\ln(L^{m})$ denotes the largest IPE value.

(5) $$IPE=-\sum_{i=1}^{L^{m}}p_{i}\ln(p_{i})/\ln(L^{m})$$

In comparison to PE and its variants, IPE preserves both absolute amplitude and temporal correlation information and utilizes a larger set of potential symbolic patterns to characterize the signal. This suggests that IPE is likely to achieve better signal differentiation. Additionally, the symbolization process described in Equations (3) and (4) minimizes the need for extensive handling of equal values in the sequence, effectively addressing the equal value issue present in PE.

### 2.3. Entropy Feature Vector Integration

Section 2.2 outlines the process for calculating IPE with a specific shape parameter $\beta_{i}$. As previously mentioned, the shape parameter determines the form of the GGD, enabling it to adapt to signals with varying distributions. In accordance with the recommendations in [34], in this paper, β is incremented from 0.1 to 2.9 in steps of 0.2. The IPE is computed for each value of β, and the resulting entropy values are combined into a 1 × 15 entropy feature vector, yielding the GGDIPE.

### 2.4. Multiscale GGDIPE

To enhance GGDIPE’s capability in handling complex time series, a multiscale version of GGDIPE is also proposed. Let $x=x_{1},x_{2},x_{3}\cdots ,x_{N}$ be the time series to be analyzed. The coarse-graining technique is employed to decompose the signal into multiple subsequences, as shown in Equation (6), where s denotes the scale factor, $r_{i}^{s}$ represents the *i*-th element of the subsequence under scale s, and $1\leq j\leq \left\lfloor N/s\right\rfloor$. It is important to note that as the scale factor increases, the length of subsequence decreases to $\left\lfloor N/s\right\rfloor$, leading to potential instability in entropy estimation. Therefore, the scale factor should be carefully chosen based on the length of the original time series being analyzed.

(6) $$r_{j}^{s}=\frac{1}{s}\sum_{i=(j-1)s+1}^{js}x_{i}$$

After coarse-graining, GGDIPE is applied to each subsequence. Assuming the maximum scale factor is $s_{\max}$, the Multiscale Generalized Gaussian Distribution Improved Permutation Entropy (MGGDIPE) is ultimately represented as a $s_{\max}\times 15$ entropy feature matrix.

## 3. Synthetic Data Analysis

In this section, the proposed GGDIPE algorithm is utilized to analyze various types of noise and chaotic signals to validate its effectiveness.

### 3.1. Noise

Noise is pervasive in practical engineering applications. The $1/f^{\alpha }$ noise has been extensively studied in previous research and is an important benchmark for evaluating the performance of entropy algorithms [2,20]. For α=0, the power spectrum density is flat across all frequencies, corresponding to white noise. For α=1 and α=2, the power of the signal decreases at varying rates as the frequency increases. Typically, 1/f noise is referred to as pink noise, while $1/f^{2}$ noise is also known as brown noise.

In this subsection, we investigate the impact of various parameters—including embedding dimension m, the number of symbols L, and data length N—on GGDIPE using three types of noise.

#### 3.1.1. Impact of Embedding Dimension

**Figure 2:**
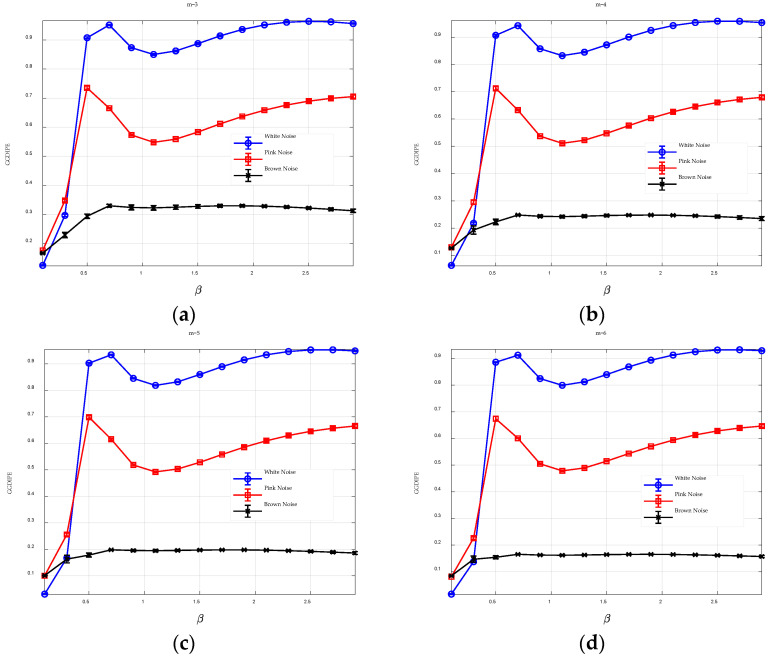
The GGDIPE analysis for three types of noise across varying embedding dimensions. (**a**) m=3 (**b**) m=4 (**c**) m=5 (**d**) m=6.

The embedding dimension determines the length of the reconstructed vectors. To explore its impact on GGDIPE, we set N=10000 and L=4, then varied m from 3 to 6, observing how the entropy changes with different values of m. In this experiment, 20 independent sets were generated for each type of noise, and the entropy analysis results are presented using error bar plots. The center of each error bar represents the mean entropy value across multiple trials, while the length of the error bar indicates the standard deviation of these results. The analysis results are shown in Figure 2.

The GGDIPE results for the three types of noise are quite similar across different embedding dimensions, with only minor variations in entropy values. For example, when m=3, the entropy values for Brown noise remain stable around 0.3 across different β values, whereas for m=6, these values slightly decrease to approximately 0.15. Furthermore, the GGDIPE characteristics for the three noise types exhibit significant differences when β exceeds 0.5, with the differences becoming more pronounced as m increases. This suggests that GGDIPE is relatively insensitive to changes in the embedding dimension, demonstrating its general applicability. In subsequent study, m=4 is selected.

As observed in Figure 2, a local maximum in entropy occurs when β is near 0.5. This is because when β≈0.5, the probability density of the generalized Gaussian distribution becomes sharply peaked, leading to the normalized values clustering around specific points. As a result, the distribution of certain patterns becomes more uniform, contributing to an increase in the entropy value.

**Figure 3:**
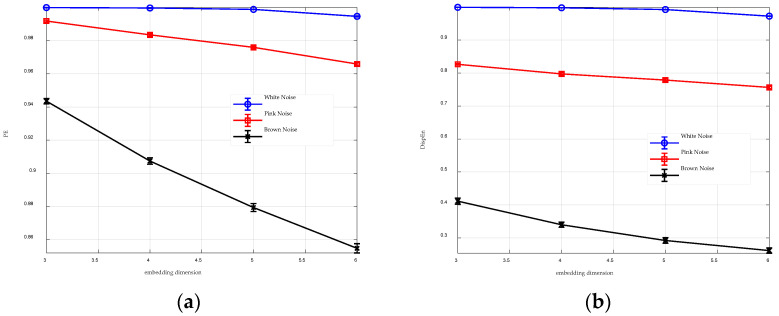
Analysis results of PE and DispEn for three types of noise across various embedding dimensions. (**a**) PE analysis result; (**b**) DispEn analysis result.

For comparison, Figure 3 presents the analysis results of PE and DispEn for three types of noise across various embedding dimensions. It appears that the embedding dimension has minimal impact on DispEn, while it significantly affects PE. Specifically, for m=3, PE assigns very high entropy values (>0.94) to all three types of noise, obscuring their differences. However, as m increases, these differences become more obvious. Thus, a larger m enhances PE’s ability to distinguish between different signals.

The comparison results between Figure 2 and Figure 3 indicate that, compared to PE, GGDIPE is more stable and less affected by changes in the embedding dimension.

#### 3.1.2. Impact of L

The parameter L defines the number of symbols used to represent the time series. To examine the effect of L on GGDIPE, we fixed m=4 and N=10000 and varied L across values of 2, 4, 6, and 8. We then observed how changes in L affect the entropy measurements. Again, for each type of noise, 20 independent trials were generated.

**Figure 4:**
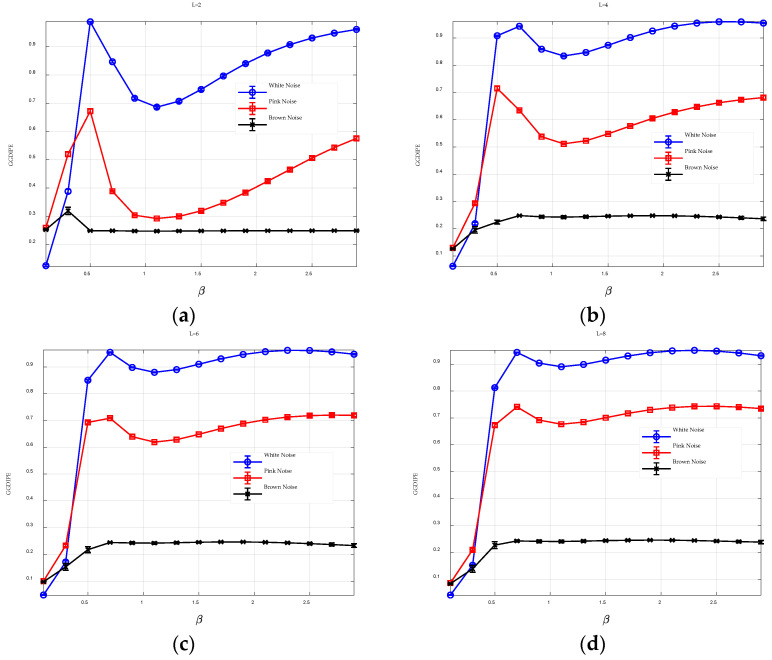
The GGDIPE analysis for three types of noise across varying L (**a**) *L* = 2; (**b**) *L* = 4; (**c**) *L* = 6; (**d**) *L* = 8.

As illustrated in Figure 4, when L=2, GGDIPE’s entropy quantification for the three types of noise exhibits significant distortion. For instance, in the case of white noise, which is inherently random and theoretically should yield the maximum entropy value, it shows an entropy value below 0.7 for certain β values, as depicted in Figure 4a.

This issue is mitigated as L increases, with the entropy value for white noise approaching 1 as L becomes larger. This improvement is attributed to the fact that a higher L reduces information loss during the symbolization process, allowing GGDIPE to capture more potential patterns of the signal and thereby enhance the accuracy of entropy estimation. Nonetheless, L should not be excessively large. As will be discussed in Section 3.2, the selection of L requires a balance between entropy estimation accuracy and noise resistance.

#### 3.1.3. Impact of Data Length

**Figure 5:**
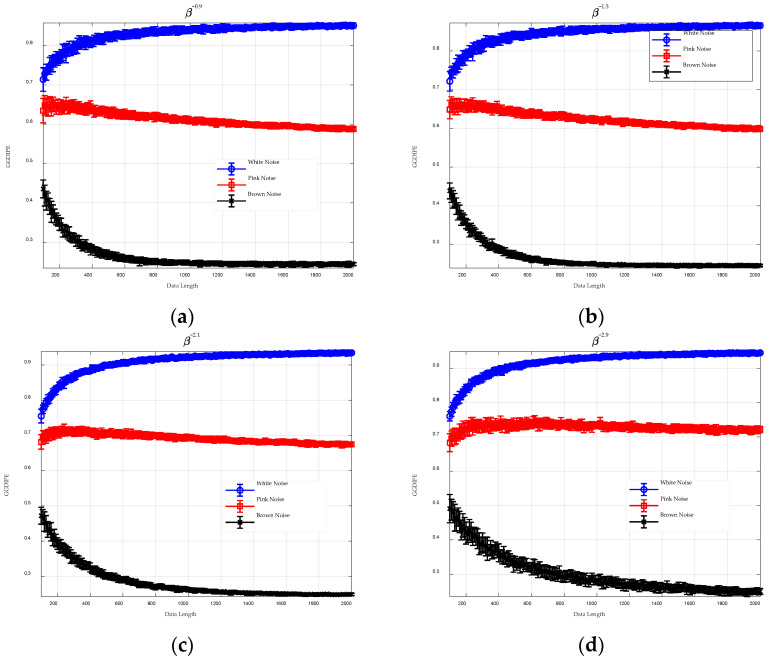
The GGDIPE analysis for three types of noise across varying data length. (**a**) β = 0.9 (**b**) β = 1.5 (**c**) β = 2.1 (**d**) β = 2.9.

To investigate the impact of data length on GGDIPE, we set m=4 and L=4 and varied N from 100 to 2000 in increments of 10. For clarity, Figure 5 displays experimental results for selected values of β. Again, for each type of noise, 20 independent trials were generated.

The results shown in Figure 5 demonstrate that GGDIPE is capable of distinguishing between the three types of noise even with relatively short data lengths, such as N=100. As the data length increases, the entropy value for white noise converges towards 1, indicating enhanced accuracy in GGDIPE’s entropy estimation. Furthermore, the ability to differentiate among the three noise types improves with longer data lengths. The GGDIPE results stabilize for all three noise types when N exceeds 1000.

**Figure 6:**
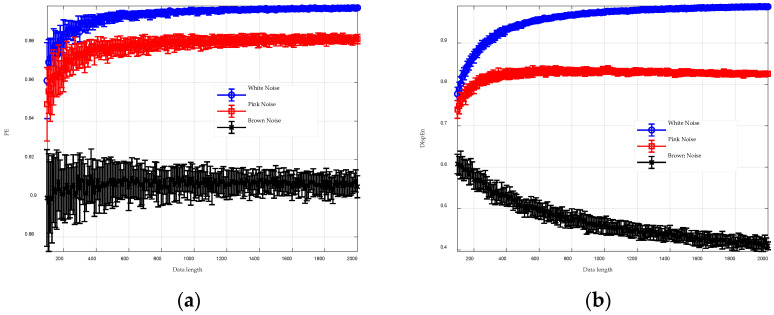
Analysis results of PE and DispEn for three types of noise across various data length. (**a**) PE analysis result; (**b**) DispEn analysis result.

For comparison, Figure 6 illustrates the experimental results for PE and DispEn. With shorter data lengths, the permutation entropy values for white noise and pink noise are very close, and the larger error bars indicate lower consistency among multiple experimental trials. As the data length increases, this issue improves; however, the permutation entropy values for all three noise types remain above 0.9, suggesting they are still quite close. DispEn generally outperforms PE, but even at a data length of 2000, the DispEn value for brown noise has not yet stabilized, and the consistency among multiple experiments is somewhat lower compared to GGDIPE.

In summary, GGDIPE exhibits superior stability with shorter data lengths compared to PE and DispEn, offering enhanced signal differentiation capabilities and demonstrating reduced sensitivity to variations in data length.

### 3.2. Noisy Lorenz Signal

To assess the noise resistance of GGDIPE, Lorenz chaotic signals were generated using Equation (7), with only the z-axis component considered. White noise of varying intensities was added to simulate different signal-to-noise ratio (SNR) conditions. For each SNR condition, 20 independent experiments were conducted. The Lorenz system was solved using a fourth-order Runge–Kutta scheme with Δt=0.001, and 10,000 data points were recorded.

(7) $$\left\{\begin{array}{l} \dot{x}=10\left(y-x\right)\\ \dot{y}=x\left(28-z\right)-y\\ \dot{z}=xy-\frac{8}{3}z \end{array}\right.$$

**Figure 7:**
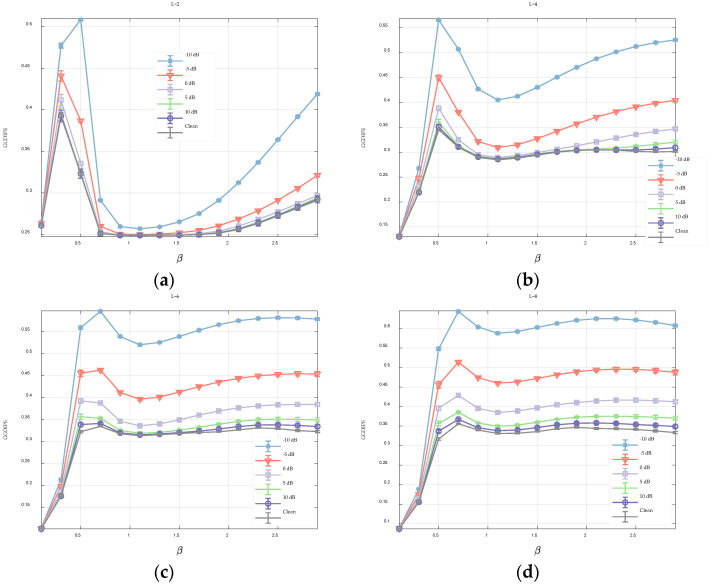
The GGDIPE analysis for noisy Lorenz signals. (**a**) L=2 (**b**) L=4 (**c**) L=6 (**d**) L=8.

Figure 7 illustrates the analysis results of GGDIPE applied to noisy Lorenz signals with varying values of L. When L=2, the results across different signal-to-noise ratio (SNR) conditions are largely consistent, with only the entropy curve for SNR = −10 dB showing a slight deviation from the clean signal’s curve. However, as L increases, these entropy curves start to diverge at higher SNR levels. For instance, when L=8, the entropy curve for 0 dB already shows a noticeable difference from that of the clean signal. As noted in Section 3.1.2, a larger L improves the accuracy of entropy estimation, which is why the GGDIPE curve for the clean Lorenz signal in Figure 7d is more precise. Comparing Figure 7a,d reveals that although the results for L=2 are more consistent across different SNRs, the accuracy of the entropy estimation is significantly reduced. This reduction in accuracy is due to the greater information loss during the symbolization process with a smaller L, despite the increased noise resistance that comes with it. Taking into account the findings from both Figure 7 and Figure 4, it is clear that the selection of L requires a balance between entropy estimation accuracy and noise resistance. In the subsequent sections, unless otherwise specified, L=4 will be used.

**Figure 8:**
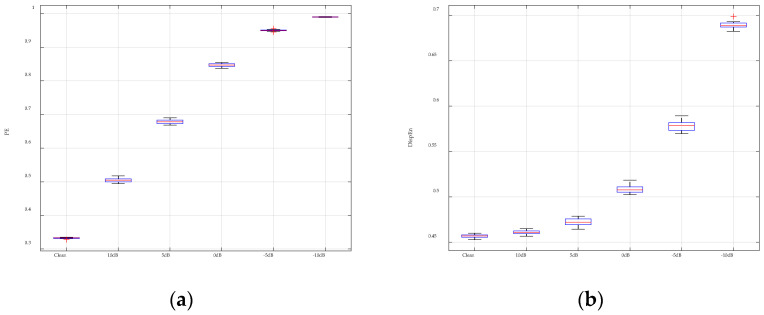
Analysis results of PE and DispEn for noisy Lorenz signals. (**a**) PE analysis result; (**b**) DispEn analysis result.

For comparison, Figure 8 presents the analysis results for PE and DispEn, displayed using box plots. The results clearly show that PE has minimal resistance to noise, with entropy values at 10 dB already significantly deviating from those of the pure Lorenz signal. In contrast, DispEn demonstrates stronger noise resistance, with noticeable deviations only occurring at 0 dB. These comparisons highlight that the proposed GGDIPE algorithm exhibits significantly better noise resistance than PE.

### 3.3. Logistic Map

**Figure 9:**
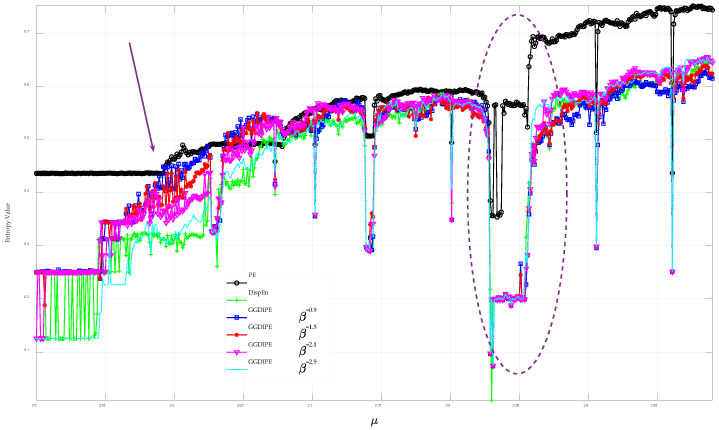
Analysis results of applying various entropy algorithms to the Logistic model.

The ability to detect periodicity and nonlinearity is a crucial metric for assessing the performance of entropy algorithms. To assess GGDIPE’s effectiveness in this area, we analyzed the Logistic model, represented by $x_{n+1}=\mu x_{n}(1-x_{n})$, where μ is a critical parameter influencing the model’s dynamic behavior. As μ varies from 3.5 to 3.99, the model exhibits period-doubling bifurcations, chaos, and intermittent periodic states, respectively. Figure 9 shows the results of applying various entropy algorithms to the Logistic model. In this analysis, μ is incremented by 0.0001 within the specified range. For each μ value, the Logistic model generates 10,000 data points, and their corresponding entropy values are calculated. For clarity, the figure displays GGDIPE results for only few specific β values.

The results show that while PE generally captures the overall dynamics of the Logistic model as μ varies, it falls short in fully describing the transition processes. In contrast, both GGDIPE and DispEn exhibit an increasing trend in the region marked by the arrow, around μ≈3.57, reflecting the system’s shift from a periodic state to chaos, consistent with the findings in [2]. Furthermore, in the elliptical region near μ≈3.84, both GGDIPE and DispEn show a marked decrease, revealing the brief periodic state in this area. These results in Figure 9 highlight GGDIPE’s strong capability in uncovering the dynamical behavior of the system.

### 3.4. Computation Cost

Computational complexity is a crucial metric for evaluating entropy algorithms, as it directly influences their suitability for real-time processing. To examine this, we analyzed 20 groups of white noise with varying data lengths using multiple entropy algorithms on the same computing device and recorded the computation time. All simulations in this section were performed using MATLAB R2024a on a computer with an i9-14900HX CPU (2.2 GHz) and 16 GB of RAM, produced by Xiaomi Communication Technology Co., LTD., in Beijing, China. For ease of comparison, the computation time for GGDIPE was recorded for a single β value. This strategy is justified because β can be selected according to specific application needs when utilizing GGDIPE.

The results in Table 1 indicate that DispEn consistently exhibits the fastest processing speed across various data lengths, with GGDIPE trailing slightly behind and PE showing the slowest processing speed. Given that PE is typically considered a real-time processing algorithm, the computational time of GGDIPE should not pose a significant barrier to its practical application.

## 4. Experimental Data Analysis

To assess the effectiveness of GGDIPE in managing complex real-world time series, the proposed algorithm was applied to a diverse set of intricate signals, including RR intervals data, EEG signals, bearing fault signals, and underwater acoustic signals.

### 4.1. RR Intervals

RR intervals are the time intervals between successive R-wave peaks in ECG signals. These intervals are crucial for analyzing heart rate variability. The experimental data used in the subsection is sourced from the Fantasia database [35], which includes data from 20 young and 20 elderly subjects. The primary objective is to determine whether the age of the subjects can be inferred from their RR interval data. Achieving this goal necessitates significant differences in the RR interval data characteristics between the young and elderly groups.

**Figure 10:**
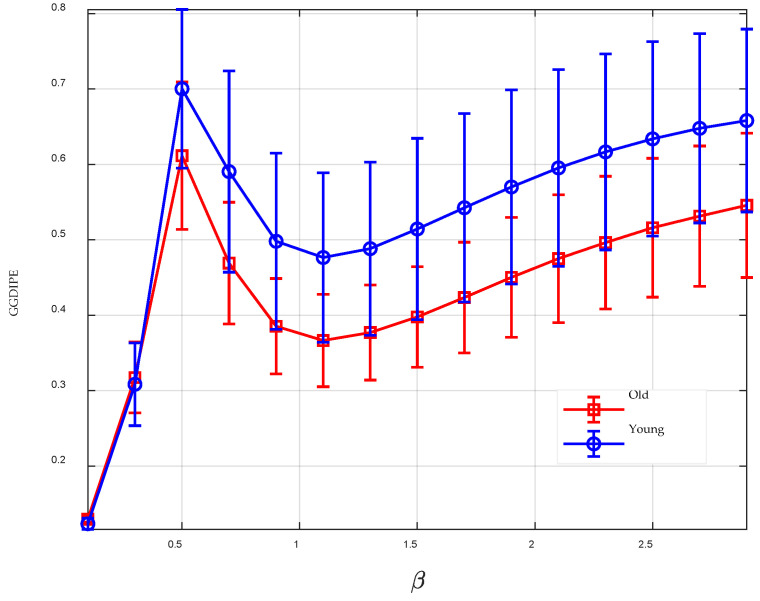
GGDIPE analysis results for the RR intervals of healthy young and healthy elderly subjects.

**Figure 11:**
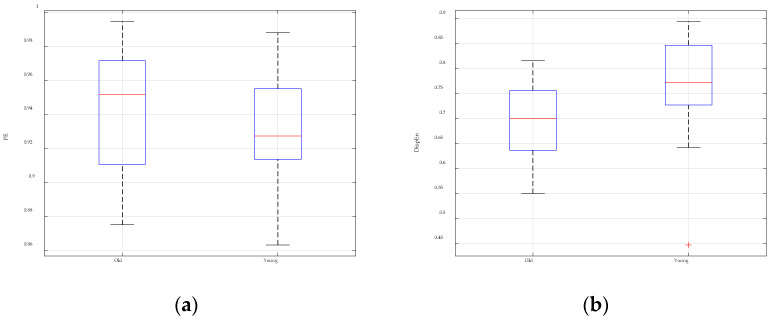
Analysis results of PE and DispEn for the RR intervals of healthy young and healthy elderly subjects. (**a**) PE analysis result; (**b**) DispEn analysis result.

The GGDIPE analysis results for the RR intervals of healthy young and elderly subjects are presented in Figure 10. The results indicate that, for most values of β, there are significant differences in the features between the RR intervals of young and elderly subjects. This demonstrates that GGDIPE is capable of effectively distinguishing age-related variations in RR interval data. For comparison, Figure 11 presents the analysis results obtained using PE and DispEn. Visually, both methods demonstrate less effective separability between the two data categories compared to GGDIPE.

To quantify the separability of the entropy features, the non-parametric Mann–Whitney U-test was applied, with the results summarized in Table 2. Due to space limitations, Table 2 displays only a subset of the *p*-values for GGDIPE under some specific β values. In the table, * indicates a *p*-value less than 0.05, ** denotes a *p*-value less than 0.01, and *** represents a *p*-value less than 0.001. It is evident that, compared to PE and DispEn, the GGDIPE features reveal more pronounced differences between the two subject groups.

### 4.2. EEG Signals

EEG signals refer to the electrical activity recorded from the brain using electroencephalography, which are widely employed in prediction of epileptic seizures. The experimental data for this section are obtained from the Bonn EEG database [36], encompassing EEG signals from four distinct subject categories: healthy subjects with eyes open (Class 0), healthy subjects with eyes closed (Class 1), subjects during epileptic interictal periods (Class 2), and subjects experiencing seizure attacks (Class 3). The data was sampled at a frequency of 173.61 Hz, with 100 segments collected for each subject group, each segment having a duration of 23.6 s and comprising 4097 sample points. The challenge is to distinguish between Class 0 and Class 1, as well as between Class 2 and Class 3. To this end, multiscale entropy analysis was applied to the data. Given that each segment contains only 4097 sample points, the maximum scale factor was set to 5 to ensure the accuracy of the multiscale entropy estimates.

**Figure 12:**
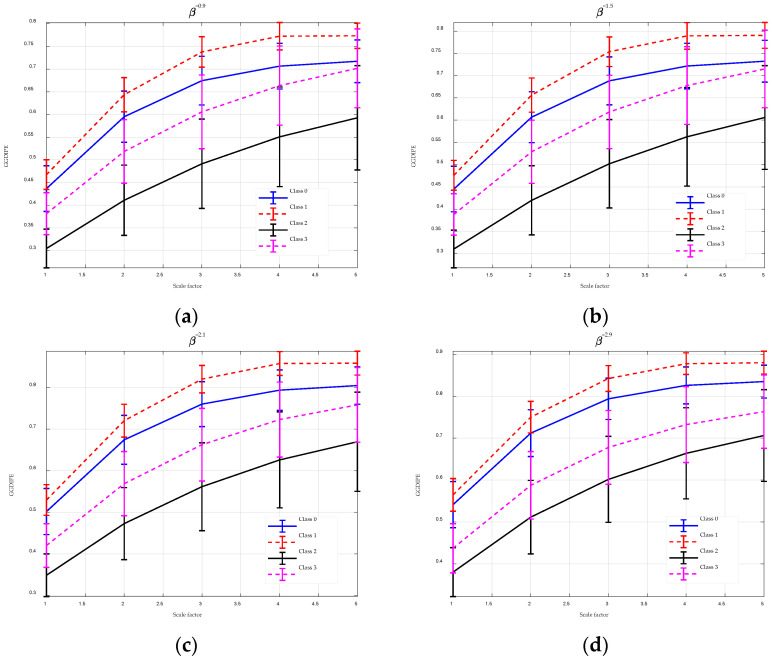
The MGGDIPE analysis for EEG signals. (**a**) β = 0.9 (**b**) β = 1.5 (**c**) β = 2.1 (**d**) β = 2.9.

**Figure 13:**
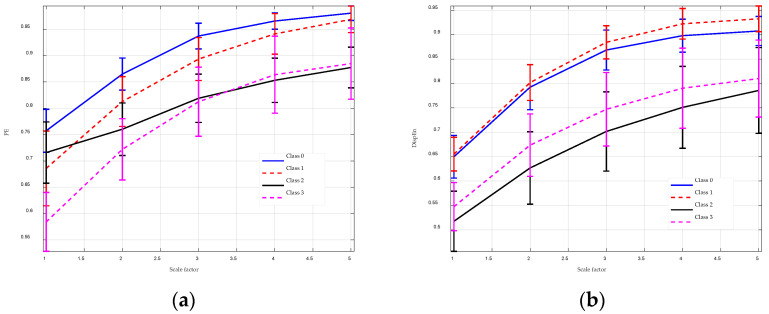
The MPE and MDE analysis for EEG signals. (**a**) MPE analysis result; (**b**) MDE analysis result.

The analysis results for MGGDIPE, MPE, and MDE are shown in Figure 12 and Figure 13, respectively. For clarity, Figure 12 presents MGGDIPE results for only a subset of β values. Across all scales, Class 1 exhibits the highest average MGGDIPE values, followed by Class 0, Class 3, and Class 2. This distinct trend is absent in the MPE and MDE results. In particular, MDE shows minimal differentiation between Class 0 and Class 1 at lower scales, while MPE values for Class 2 and Class 3 nearly overlap at higher scales. Visually, MGGDIPE provides markedly better discrimination among the different signal groups compared to MPE and MDE.

To further substantiate this observation, the non-parametric Mann–Whitney U-test was applied to quantify the significance of differences between the feature sets, with the results detailed in Table 3, Table 4, Table 5, Table 6, Table 7 and Table 8. The results are highly consistent with the feature extraction outcomes. Across all scales, the *p*-values for MGGDIPE are less than 0.001, indicating significant differences between the MGGDIPE features of class 0 versus class 1 and class 2 versus class 3. In contrast, MPE and MDE exhibit significant differences only at specific scales. These results suggest that MGGDIPE provides superior discriminative capability for differentiating between various EEG signal types compared to MPE and MDE.

### 4.3. Bearing Fault Signals

Bearings are essential components in many mechanical systems, and failures in bearings can lead to significant operational disruptions and severe consequences. Therefore, the early detection of bearing faults is crucial and presents a substantial challenge in the industrial sector. This section assesses the effectiveness of MGGDIPE for bearing fault diagnosis using data from the Jiangnan University Database [37,38]. The database includes four types of signals: inner-race defect, outer-race defect, rolling element defect, and normal signals. Data were collected at three different rotational speeds—600, 800, and 1000 rpm—with a sampling frequency of 50 kHz. Each signal type was recorded for 10 s at each speed. Due to the long duration of the signals, each dataset was segmented into non-overlapping chunks with 10,000 samples, and the maximum scale factor was set to 10.

**Figure 14:**
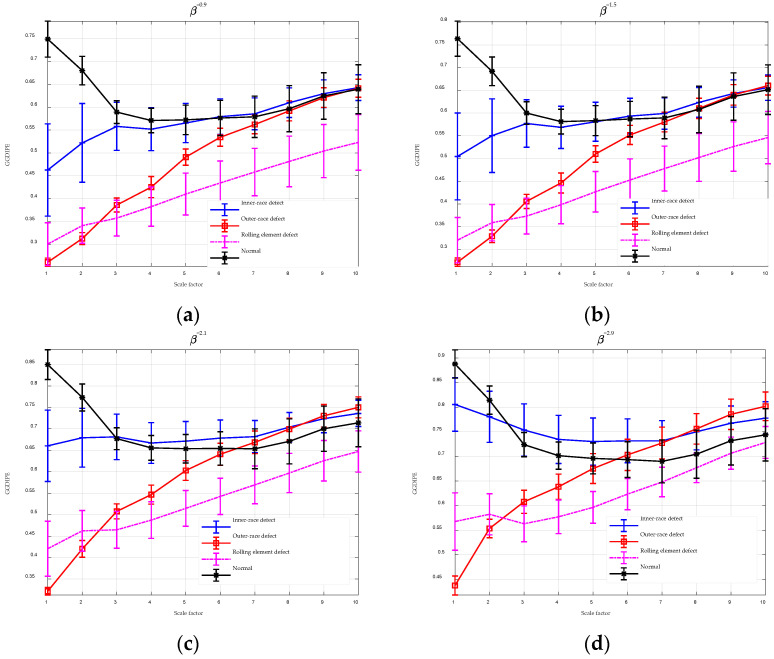
The MGGDIPE analysis for bearing fault signals. (**a**) β = 0.9 (**b**) β = 1.5 (**c**) β = 2.1 (**d**) β = 2.9.

**Figure 15:**
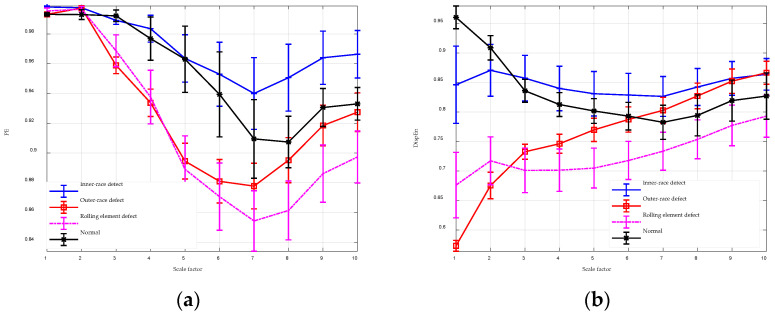
The MPE and MDE analysis for bearing fault signals. (**a**) MPE analysis result; (**b**) MDE analysis result.

The analysis results for MGGDIPE, MPE, and MDE are shown in Figure 14 and Figure 15, respectively. For clarity, Figure 14 presents MGGDIPE results for only a subset of β values. The MGGDIPE features demonstrate clear discriminative ability among the four signal types at low scales, with similar patterns observed in the MDE analysis. In contrast, MPE features for the four signal types exhibit substantial overlap at low scales, though their discriminative power improves with increasing scale factors. Nevertheless, MPE values for all signal types remain clustered between 0.84 and 0.98, reflecting weaker separability compared to MDE and MGGDIPE. These findings indicate that MGGDIPE has significant potential for effective bearing fault diagnosis.

### 4.4. Underwater Acoustic Signals

Underwater acoustic signals refer to sound waves transmitted through water. The ocean ambient noise and ship-radiated noise are the most frequently studied signals. This section assesses the effectiveness of MGGDIPE in classifying underwater acoustic signals using data from the ShipsEar database [39]. The dataset, sampled at 52,734 Hz, comprises four signal categories: Passenger, Ocean liner, Motorboat, and Ambient noise, with each category including recordings from multiple vessels. In contrast to some prior studies, this analysis requires feature extraction and identification from a larger number of targets, which presents a greater challenge. To facilitate analysis, the dataset was divided into numerous segments, each lasting 3 s. A comprehensive description of the signals is provided in Table 9.

**Figure 16:**
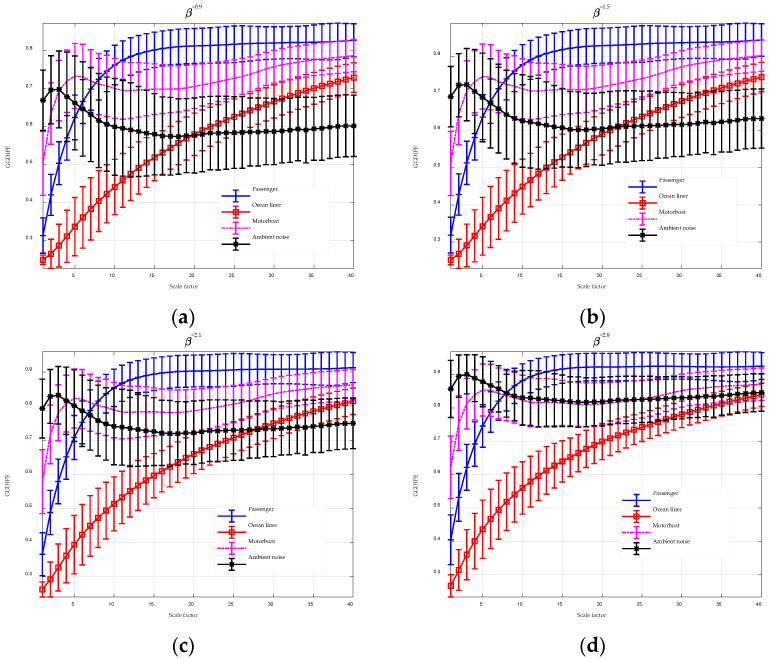
The MGGDIPE analysis for underwater acoustic signals. (**a**) β = 0.9 (**b**) β = 1.5 (**c**) β = 2.1 (**d**) β = 2.9.

**Figure 17:**
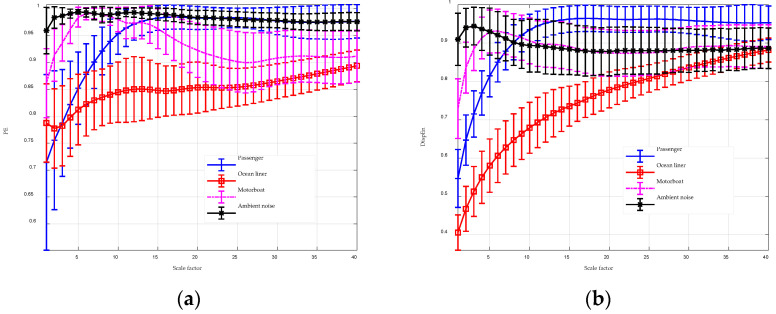
The MPE and MDE analysis for underwater acoustic signals. (**a**) MPE analysis result; (**b**) MDE analysis result.

The analysis results for MGGDIPE, MPE, and MDE are shown in Figure 16 and Figure 17, respectively. For clarity, Figure 16 presents MGGDIPE results for only a subset of β values. Given the extended length of the data, a larger maximum scale factor was chosen to capture a broader range of multiscale features, with a value of 40 applied in this section. The results in Figure 16 illustrate the advantages of using GGDCDF for data normalization. When β is large (e.g., 2.1 and 2.9), the entropy values of Motorboat and Ambient noise overlap at higher scales. However, with smaller β values (e.g., 0.9 and 1.5), the entropy values of these two signal types show clear separability at higher scales. This demonstrates that adjusting β enhances the feature extraction capabilities of GGDIPE. Additionally, the MGGDIPE feature trends, which describe how entropy varies with the scale factor, demonstrate distinct separability across the four types of underwater acoustic signals. As the scale factor increases from 1 to 40, the entropy of the Ocean liner steadily rises from 0.25 to 0.7, while the Passenger shows a rapid increase from 0.31 to 0.8, stabilizing after scale 20. The entropy of Ambient noise gradually decreases from approximately 0.7 to 0.6, leveling off after scale 15. In contrast, the entropy of the Motorboat signal increases sharply from 0.5 to 0.72, experiences a slight dip around scale 15, and then gradually ascends to 0.8.

In Figure 17a, it is evident that the separability of MPE features for the four types of underwater acoustic signals is significantly weaker than that of MGGDIPE. This is partly due to PE’s omission of signal amplitude information and its relatively poor noise resistance. In Figure 17b, the feature extraction results of MDE closely resemble those shown in Figure 16d, indicating consistency between the two methods. However, due to the absence of the β parameter, MDE exhibits weaker discrimination between Motorboat and Ambient noise compared to MGGDIPE.

To further quantitatively compare the effectiveness of the three multiscale entropy algorithms in underwater target classification, a probabilistic neural network (PNN) was used to train and test the extracted features, with recognition rate serving as the performance metric. The training and testing samples were non-overlapping, with 100 randomly selected segments from each signal type used for testing, while the remaining segments were used for training. The recognition results are presented in Table 10, Table 11 and Table 12. To highlight the impact of the parameter β on data classification, the classification results of MGGDIPE at specific β values are also provided in Appendix A.

The recognition results align closely with the previous analysis, with MGGDIPE achieving an accuracy rate of 97.5%, substantially exceeding those of MDE (70.5%) and MPE (62.5%). These findings underscore the significant potential of the proposed MGGDIPE algorithm for underwater target recognition.

## 5. Conclusions

To enhance the performance of entropy algorithms in analyzing complex time series, this paper introduces the GGDIPE algorithm and its multiscale variant. GGDIPE employs the GGDCDF for data normalization, making it versatile across various distributions. The algorithm further processes the normalized data with the IPE approach, which maintains both the absolute magnitude and temporal correlations of the signals, overcoming the equal value issue found in traditional PE. Simulation results indicate that GGDIPE is less sensitive to parameter variations, exhibits strong noise resistance, accurately reveals the dynamic behavior of chaotic systems, and operates significantly faster than PE, with speed comparable to DispEn. Real-world data analysis shows that MGGDIPE provides markedly better separability for RR interval signals, EEG signals, bearing fault signals, and underwater acoustic signals compared to conventional MPE and the recently proposed MDE algorithm. Notably, in underwater target recognition tasks, MGGDIPE achieved a classification accuracy of 97.5% across four types of acoustic signals, substantially surpassing the performance of MDE (70.5%) and MPE (62.5%). Thus, the proposed method demonstrates exceptional capability in processing complex time series data.

## Figures and Tables

**Table 1:** The computation time required by different entropy algorithms to complete 20 sets of noise analysis experiments.

	*N* = 10,000	*N* = 50,000	*N* = 100,000
**PE**	0.43 s	2.04 s	4.01 s
**DispEn**	0.13 s	0.30 s	0.51 s
**GGDIPE**	0.13 s	0.39 s	0.69 s

**Table 2:** The non-parametric Mann–Whitney U-test results for the RR intervals of healthy young and healthy elderly subjects. * indicates a *p*-value less than 0.05, ** denotes a *p*-value less than 0.01, and *** represents a *p*-value less than 0.001.

	β = 0.9	β = 1.5	β = 2.1	β = 2.9	PE	DispEn
*p*-values	$2.34\times 10^{-4}$ ***	$2.61\times 10^{-4}$ ***	$5.35\times 10^{-4}$ ***	0.0019 **	0.2792	0.0038 *

**Table 3:** The significance of differences in MGGDIPE (β=0.9) features between distinct categories. *** denotes *p*-values less than 0.001.

	Scale 1	Scale 2	Scale 3	Scale 4	Scale 5
Class0 vs. Class1	$p=4.575\times 10^{-8}$ ***	$p=7.869\times 10^{-11}$ ***	$p=3.026\times 10^{-17}$ ***	$p=4.815\times 10^{-21}$ ***	$p=3.922\times 10^{-19}$ ***
Class2 vs. Class3	$p=1.839\times 10^{-22}$ ***	$p=7.352\times 10^{-18}$ ***	$p=2.370\times 10^{-15}$ ***	$p=3.923\times 10^{-14}$ ***	$p=8.281\times 10^{-14}$ ***

**Table 4:** The significance of differences in MGGDIPE (β=1.5) features between distinct categories. *** denotes *p*-values less than 0.001.

	Scale 1	Scale 2	Scale 3	Scale 4	Scale 5
Class0 vs. Class1	$p=5.857\times 10^{-8}$ ***	$p=6.063\times 10^{-11}$ ***	$p=8.717\times 10^{-18}$ ***	$p=2.687\times 10^{-21}$ ***	$p=9.414\times 10^{-20}$ ***
Class2 vs. Class3	$p=1.250\times 10^{-22}$ ***	$p=6.322\times 10^{-18}$ ***	$p=2.667\times 10^{-15}$ ***	$p=3.639\times 10^{-14}$ ***	$p=1.551\times 10^{-13}$ ***

**Table 5:** The significance of differences in MGGDIPE (β=2.1) features between distinct categories. *** denotes *p*-values less than 0.001.

	Scale 1	Scale 2	Scale 3	Scale 4	Scale 5
Class0 vs. Class1	$p=3.076\times 10^{-6}$ ***	$p=1.552\times 10^{-9}$ ***	$p=5.904\times 10^{-16}$ ***	$p=3.395\times 10^{-21}$ ***	$p=1.817\times 10^{-18}$ ***
Class2 vs. Class3	$p=2.688\times 10^{-16}$ ***	$p=1.322\times 10^{-12}$ ***	$p=5.496\times 10^{-11}$ ***	$p=6.388\times 10^{-10}$ ***	$p=3.699\times 10^{-9}$ ***

**Table 6:** The significance of differences in MGGDIPE (β=2.9) features between distinct categories. *** denotes *p*-values less than 0.001.

	Scale 1	Scale 2	Scale 3	Scale 4	Scale 5
Class0 vs. Class1	$p=4.966\times 10^{-5}$ ***	$p=5.857\times 10^{-8}$ ***	$p=3.047\times 10^{-13}$ ***	$p=3.651\times 10^{-17}$ ***	$p=6.527\times 10^{-16}$ ***
Class2 vs. Class3	$p=2.402\times 10^{-9}$ ***	$p=2.125\times 10^{-8}$ ***	$p=2.545\times 10^{-7}$ ***	$p=8.088\times 10^{-6}$ ***	$p=1.592\times 10^{-4}$ ***

**Table 7:** The significance of differences in MPE features between distinct categories. *, and *** denotes *p*-values less than 0.05 and 0.001, respectively.

	Scale 1	Scale 2	Scale 3	Scale 4	Scale 5
Class0 vs. Class1	$p=7.006\times 10^{-14}$ ***	$p=1.841\times 10^{-14}$ ***	$p=2.313\times 10^{-14}$ ***	$p=5.988\times 10^{-5}$ ***	p=0.0118 *
Class2 vs. Class3	$p=4.7184\times 10^{-29}$ ***	$p=9.922\times 10^{-6}$ ***	p=0.9932	p=0.0925	p=0.1484

**Table 8:** The significance of differences in MDE features between distinct categories. **, *** denotes *p*-values less than 0.01, and 0.001, respectively.

	Scale 1	Scale 2	Scale 3	Scale 4	Scale 5
Class0 vs. Class1	p=0.1582	p=0.0739	p=0.0043 **	$p=6.173\times 10^{-7}$ ***	$p=4.288\times 10^{-9}$ ***
Class2 vs. Class3	p=0.0025 **	$p=6.438\times 10^{-5}$ ***	$p=8.265\times 10^{-4}$ ***	p=0.0081 **	p=0.1314

**Table 9:** Detailed information of underwater acoustic signals.

Categories	Ship Name	Number of Segments
Passenger	Mar de Cangas	267
	Mar de Onza	124
	Pirata de Salvora	65
	Arrois	103
Ocean liner	MSC Opera	160
	Adventure of the sea	89
	Costa Voyager	397
Motorboat	Small Yacht	76
	Motorboat2	86
	High speed motorboat	36
	Zodiac	96
Ambient noise	\	267

**Table 10:** PNN recognition results for MGGDIPE.

Categories	Recognized As				Classification Accuracy
	Passenger	Ocean Liner	Motorboat	Ambient Noise	
Passenger	100	0	0	0	100%
Ocean liner	0	100	0	0	100%
Motorboat	1	6	90	3	90%
Ambient noise	0	0	0	100	100%
In total	\	\	\	\	97.5%

**Table 11:** PNN recognition results for MPE.

Categories	Recognized As				Classification Accuracy
	Passenger	Ocean Liner	Motorboat	Ambient Noise	
Passenger	48	0	0	52	48%
Ocean liner	19	81	0	0	81%
Motorboat	0	4	21	75	21%
Ambient noise	0	0	0	100	100%
In total	\	\	\	\	62.5%

**Table 12:** PNN recognition results for MDE.

Categories	Recognized As				Classification Accuracy
	Passenger	Ocean Liner	Motorboat	Ambient Noise	
Passenger	100	0	0	0	100%
Ocean liner	19	81	0	0	81%
Motorboat	6	4	21	69	21%
Ambient noise	0	0	20	80	80%
In total	\	\	\	\	70.5%

**Figure A1:**
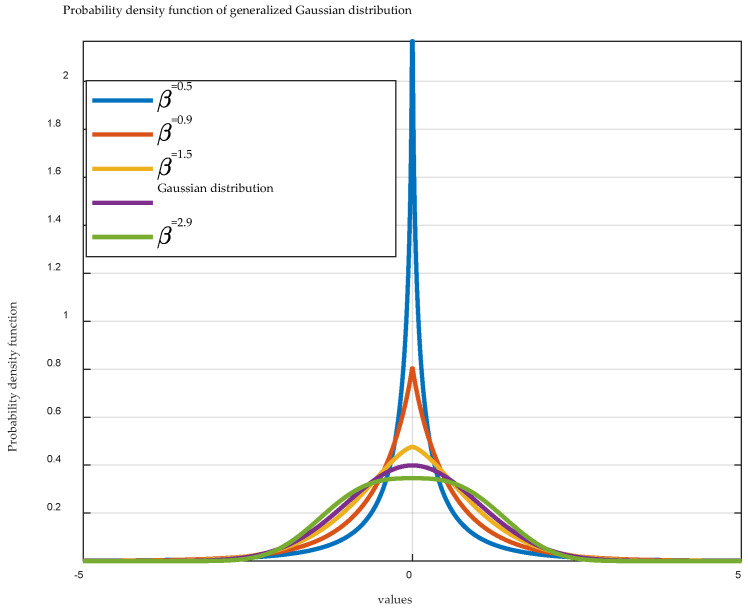
Probability density function of generalized Gaussian distribution.

## Data Availability

The data used to support the findings of this study are available from the corresponding author upon request.

## Appendix A

To better understand the algorithm proposed in this paper, this appendix provides an explanation through a simple example. Supposing that the signal to be analyzed is x=[1.5,−1.06,0.57,−2.21,0.43,−0.7,−0.74,0.32,1.5,−1.2] and β=0.9, Equation (1) transforms x into y=[0.8730, 0.2979, 0.6558, 0.0726, 0.6186, 0.3937, 0.3834, 0.5907, 0.8730, 0.2611]. Setting m=4 and L=4, phase space reconstruction results can be expressed as Equation (A1), and symbolization results can be obtained through Equations (3) and (4), as shown in Equation (A2). Obviously, $p_{4231}=p_{2313}=p_{3132}=p_{1322}=p_{3223}=p_{2224}=p_{2342}=\frac{1}{7}$, hence $IPE=\frac{-\sum_{i=1}^{7}\frac{1}{7}\ln(\frac{1}{7})}{\ln(4^{4})}$ = 0.3510.

(A1) $$\left[\begin{array}{cccc} 1.5 & -1.06 & 0.57 & -2.21\\ -1.06 & 0.57 & -2.21 & 0.43\\ 0.57 & -2.21 & 0.43 & -0.7\\ -2.21 & 0.43 & -0.7 & -0.74\\ 0.43 & -0.7 & -0.74 & 0.32\\ -0.7 & -0.74 & 0.32 & 1.5\\ -0.74 & 0.32 & 1.5 & -1.2 \end{array}\right]$$

(A2) $$\left[\begin{array}{cccc} 4 & 2 & 3 & 1\\ 2 & 3 & 1 & 3\\ 3 & 1 & 3 & 2\\ 1 & 3 & 2 & 2\\ 3 & 2 & 2 & 3\\ 2 & 2 & 2 & 4\\ 2 & 3 & 4 & 2 \end{array}\right]$$

## Appendix B

The probability density function of the generalized Gaussian distribution is given by Equation (A3), where μ and σ denote the mean and the standard deviation of the distribution, Γ stands for the gamma function, and $\rho =\sigma \sqrt{\left(\Gamma (1/\beta )/\Gamma (3/\beta )\right)}$. Figure A1 shows the probability density function of the generalized Gaussian distribution and the Gaussian distribution.

(A3) $$f(x;\mu ,\rho ,\beta )=\frac{\beta }{2\rho \Gamma (1/\beta )}\exp\left(-{\left(\frac{\left|x-\mu \right|}{\rho }\right)}^{\beta }\right)$$

## Appendix C

To demonstrate the impact of the parameter β on data classification, the classification results of MGGDIPE at specific β values are provided in Table A1 and Table A2.

The results indicate that utilizing a single β value for data normalization significantly decreases the recognition rate of Motorboat. Specifically, when β=2.1, the normalization closely approximates the cumulative distribution function of the normal distribution, yielding a recognition rate of 74% for Motorboat. In contrast, using β=1.5 improves the recognition rate to 82%. This finding highlights the considerable impact of different normalization methods on recognition accuracy. In the absence of prior knowledge about the true distribution of the experimental data, employing multiple functions for data normalization is a prudent approach that aligns with the principles underlying the proposed GGDIPE method. Furthermore, the results presented in Table 10 demonstrate that combining multiscale entropy across various β values into a feature vector enhances data separability.

**Table A1:** PNN recognition results for MGGDIPE with β=1.5.

Categories	Recognized As				Classification Accuracy
	Passenger	Ocean Liner	Motorboat	Ambient Noise	
Passenger	100	0	0	0	100%
Ocean liner	0	100	0	0	100%
Motorboat	13	5	82	0	82%
Ambient noise	0	0	4	96	96%
In total	\	\	\	\	94.5%

**Table A2:** PNN recognition results for MGGDIPE with β=2.1.

Categories	Recognized As				Classification Accuracy
	Passenger	Ocean Liner	Motorboat	Ambient Noise	
Passenger	100	0	0	0	100%
Ocean liner	0	100	0	0	100%
Motorboat	20	4	74	2	74%
Ambient noise	0	0	2	98	98%
In total	\	\	\	\	93%
